# Circulating *EGFR* Mutations in Patients with Lung Adenocarcinoma by Circulating Tumor Cell Isolation Systems: A Concordance Study

**DOI:** 10.3390/ijms231810661

**Published:** 2022-09-13

**Authors:** Shih-Hong Li, Min-Hsien Wu, Hung-Ming Wang, Ping-Chih Hsu, Yueh-Fu Fang, Chih-Liang Wang, Hui-Chun Chu, Hung-Chih Lin, Li-Yu Lee, Ching-Yang Wu, Cheng-Ta Yang, Jen-Shi Chen, Jason Chia-Hsun Hsieh

**Affiliations:** 1 Division of Thoracic Medicine, Department of Internal Medicine, Saint Paul’s Hospital, Taoyuan 33069, Taiwan; 2 Division of Thoracic Medicine, Department of Internal Medicine, Chang Gung Memorial Hospital at Linkou, Taoyuan City 33305, Taiwan; 3 College of Medicine, Chang Gung University, Taoyuan City 33302, Taiwan; 4 Tissue Engineering and Microfluidic Biochip Lab, Graduate Institute of Biomedical Engineering, Chang Gung University, Taoyuan City 33302, Taiwan; 5 Circulating Tumor Cell Lab, Division of Hematology-Oncology, Department of Internal Medicine, Chang Gung Memorial Hospital at Linkou, Taoyuan City 33305, Taiwan; 6 Department of Pathology, Chang Gung, Memorial Hospital at Linkou, Taoyuan City 33305, Taiwan; 7 Thoracic and Cardiovascular Surgery Division, Department of Surgery, Chang Gung Memorial Hospital, Chang Gung University, Taoyuan City 33305, Taiwan; 8 Division of Hematology-Oncology, Department of Internal Medicine, New Taipei City Municipal Tucheng Hospital, New Taipei 23652, Taiwan

**Keywords:** liquid biopsy, circulating tumor cells, *EGFR* mutation, lung adenocarcinoma

## Abstract

**Background:** We developed a hybrid platform using a negative combined with a positive selection strategy to capture circulating tumor cells (CTCs) and detect epidermal growth factor receptor (*EGFR*) mutations in patients with metastatic lung adenocarcinoma. **Methods:** Blood samples were collected from patients with pathology-proven treatment-naïve stage IV lung adenocarcinoma. Genomic DNA was extracted from CTCs collected for *EGFR* mutational tests. The second set of CTC-EGFR mutational tests were performed after three months of anti-cancer therapy. **Results:** A total of 80 samples collected from 28 patients enrolled between July 2016 and August 2018. Seventeen patients had *EGFR* mutations, including Exon 19 deletion (n = 11), *L858R* (n = 5), and de-novo *T790* and *L858R* (n = 1). Concordance between tissue and CTCs before treatment was 88.2% in *EGFR*- mutant patients and 90.9% in non-mutant patients. The accuracy, sensitivity, specificity, positive predictive value, and negative predictive value of *EGFR* mutation tests for CTCs were 89.3%, 88.2%, 90.9%, 93.8%, and 83.3%, respectively. **Conclusions**: CTCs captured by a hybrid platform using a negative and positive selection strategy may serve as a suitable and reliable source of lung cancer tumor DNA for detecting *EGFR* mutations, including T790M.

## 1. Introduction

Lung cancer is one of the most lethal malignancies among all cancer types worldwide [1], with non-small cell lung cancers (NSCLC) accounting for ~85% of lung cancers, including adenocarcinoma, squamous cell carcinoma, large cell carcinoma, and the other histological types. With the increasing improvement in molecular science, many NSCLC driver genes, including epidermal growth factor receptor (*EGFR*), *EML4-ALK* fusion, *ROS1*, *RET*, and *MET*, have been recognized and have responded to new target therapies in this decade [2].

The most common driver gene mutation in NSCLC is *EGFR*, which has been reported to have a high incidence in the East Asian population [3,4]. In 2010, the first EGFR tyrosine kinase inhibitor (TKI), Gefitinib, had an excellent response rate and progression-free survival (PFS) compared to chemotherapy for patients with mutant *EGFR* advanced-stage NSCLC [5]. Since more EGFR TKIs, including Erlotinib, Afatinib, Dacomitinib, and Osimertinib, have been developed in this decade, the survival rate of NSCLC patients has greatly improved in recent years worldwide [6,7].

However, most patients with NSCLC have progressive disease after 9–13 months of first-line EGFR TKI treatment due to new resistant driver genes (T790M) or other resistant mechanisms [8,9]. To evaluate resistance mechanisms, repeated biopsies are performed as the disease progresses. Obtaining molecular information is an important aspect in clinical practice. However, owing to increased disease severity and poor performance, some patients have contraindications or intolerance to the invasive procedures required for biopsy [10], and a safe and convenient method needs to be developed to overcome it. Thus, the capture of tumoral genomic content in the blood, called a “liquid biopsy”, has been developed as an alternative to tissue biopsy [11,12].

The liquid biopsy is a less-invasive tool for detecting resistant genes during targeted therapy or monitoring tumor-related DNA alterations [13,14,15,16]. Among the kinds of liquid biopsies, circulating tumor DNA (ctDNA) and circulating tumor cells (CTCs) are the most widely discussed [12]. The ctDNA are short fragments of double-stranded DNA that are shed from tumors during necrosis or apoptosis either actively or passively [17]. However, ctDNA only exists in a very small portion of total circulating DNA, resulting in an extremely high cost to detect a low proportion of tumor mutations in peripheral blood samples [18]. CTCs have detached from the primary tumor and metastatic sites and are shed in the bloodstream. Several different methods have been developed to enrich and capture CTCs [19,20]. There are two main methods to isolate CTCs: (1) a label-dependent method, based on the antibody to detect specific surface markers of the CTCs or WBCs, and (2) a label-independent method, based on the physical or biological properties of the CTCs, such as size [21]. The label-dependent method has two selection methods: (1) positive selection, targeting tumor-specific antigens expressed by CTCs, such as epithelial cell adhesion molecules (Ep-CAM), mucin1 (MUC1), or human epidermal growth factor receptor 2 (HER2); and (2) negative selection, targeting antigens expressed by background cells but not by CTCs to remove them, such as CD45 [22]. Some evidence has suggested that high-purity CTC could guide and monitor the results of cancer therapy, including lung cancer [21,23,24,25,26]. To obtain DNA signals from CTCs (not from plasma), processes or devices to obtain high-purity, non-damaged, living CTCs, are required. To achieve the high-quality cell requirements, using a microfluidic chip-based CTC isolation protocol to achieve cell-friendly and high-purity CTC isolation for standard real-time polymerase chain reaction (RT-PCR) is reasonable [14,27]. Clinicians can quickly obtain *EGFR* mutation results using this method when dealing with a patient newly diagnosed with lung cancer.

In this study, we aimed to (i) propose an efficient CTC isolation protocol for precise *EGFR* mutation testing; and then (ii) compare the mutational analysis between cancer tissues and CTCs. We hypothesized that CTC-*EGFR* mutation detection can provide a quicker method of identification so that clinicians can promptly use anti-EGFR inhibitors.

## 2. Results

### 2.1. Patient Enrollment and Study Flow

We prospectively enrolled 28 patients diagnosed with metastatic lung adenocarcinoma between August 2016 and August 2018. Table 1 briefly demonstrates the basic characteristics of the enrolled patients. Among the enrolled patients, 12 (42.9%) had stage Iva disease and 16 (57.1%) had stage IVb disease (AJCC 8th edition). The most common metastatic sites were the contralateral lung (lung-to-lung metastasis, M1a, n = 14, 50.0%) and bone metastasis (n = 13, 46.4%). The EGFR mutation from the tissue showed 11 non-mutant (39.3%) and exon 19 deletion (n = 11, 39.3%), L858R (n = 5, 17.9%) and synchronous de novo T790 and L858R (n = 1, 3.6%).

**Figure 1:**
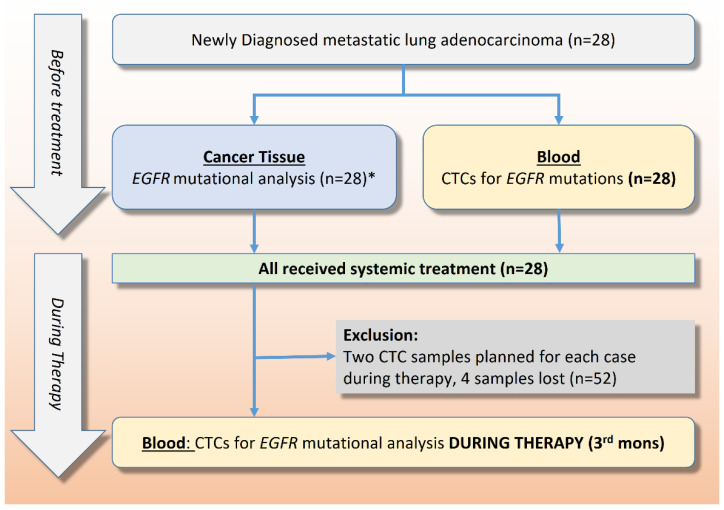
**Study flow** (Abbreviations: EGFR, epidermal growth factor receptor; CTC, circulating tumor cells; PS-CTC, positively selected circulating tumor cells; WGA, whole-genome amplification; ARMS, amplification refractory mutation system, Seq., sequencing). * The results of cancer tissue EGFR mutational analysis were *EGFR*^mutant^ (n = 17) and *EGFR^non-mutant type^* (n = 11), which were unknown before CTC isolation.

**Figure 2:**
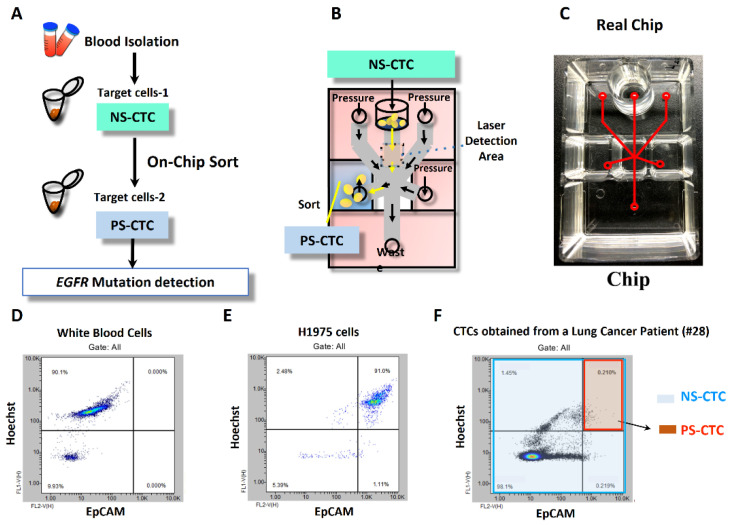
Common protocol for isolating circulating tumor cells and the confirmatory analysis by flow cytometry. (**A**) The workflow of negatively selected circulating tumor cells (NS-CTCs) systems and positively selected circulating tumor cells (PS-CTCs) systems for gene mutation identification. (**B**,**C**) The illustration of the On-Chip Sort sorting mechanism followed the intra-chamber pressure guidance for sorting the EpCAM^+^ Hoechst^+^ cells for positive selection-based enrichment. Flow-based analysis of the population of EpCAM^+^ cells in three different types of nucleated cells by On-Chip Sort, such as (**D**) human white blood cells (WBCs), EpCAM-lower expression. (**E**) Human lung adenocarcinoma cell line (H1975 cells), EpCAM-higher expression. (**F**) CTCs of a representative patient with lung adenocarcinoma (#28), the sub-population of negative selection (blue box) and positive selection (red box)-based CTCs for following each separating system.

**Figure 3:**
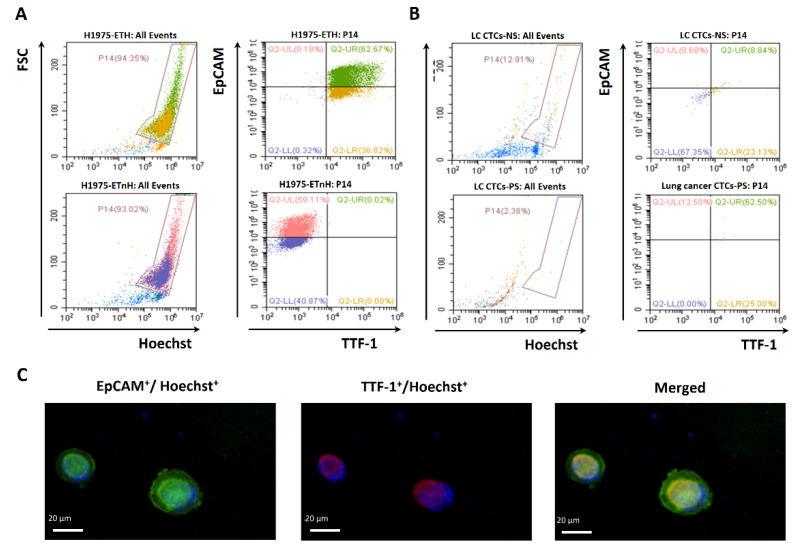
Demonstration of lung circulating tumor cells (CTCs) identified from a sample with lung cancer by thyroid transcription factor-1 (TTF-1). (**A**) Flow cytometer analysis of CTCs of a sample with lung cancer for the EpCAM^+^ and TTF-1^+^ in the Hoechst^+^ population. (**B**) The EpCAM^+^/TTF-1^+^/Hoechst^+^ cells collected from On-Chip Sort were confirmed by confocal microscopy (scale bar = 10 μm). TTF-1, Thyroid transcription factor-1. (**C**) Immunofluorescence stainings demonstrate two typical CTCs expressing EpCAM, TTF-1, and Hoechst from a lung cancer patient.

Figure 1 demonstrates the study flow of this prospective trial. We collected 80 CTC samples during the study period, including 28 before first-line therapy (baseline) and 52 CTC samples after 3 months of systemic treatments. Four CTC specimens were missed (dropped out rate: 4/80 = 5%) because of two cases with failure to follow-up and two withdrawals. Representative CTC isolation before EGFR mutational testing is shown with flow gating strategies in Figure 2A–F. Microscopic fluorescence confirmation for CTCs expressing EpCAM and TTF-1, captured from an actual lung cancer patient, is shown in Figure 3A–C.

### 2.2. Concordance of EGFR Mutational Status between CTCs and Tissues

To evaluate the concordance between *EGFR* mutations in cancer tissue and CTC samples, we separately analyzed the samples (n = 28) before systemic therapy from those during therapy (n = 52, 3rd month after treatment initiation). Table 2 displays the results of *EGFR* testing of cancer tissue and CTC samples. In the group before therapy (n = 28), the concordance rates were 88.2% and 90.9% in mutant *EGFR* and non-mutant *EGFR* groups, respectively. In the group under treatment (n = 52), the concordance rate between CTC and tissue was 95.5% for non-mutant *EGFR*. (Table 2A). Overall, the accuracy, sensitivity, specificity, positive predictive value, and negative predictive value of *EGFR* mutation tests for CTCs were 89.3%, 88.2%, 90.9%, 93.8%, and 83.3%, respectively.

To improve the efficiency and accuracy, 12 peripheral CTC samples were subjected to ARMS-PCR together with the original protocol for comparison during the trial. The results are presented in Table 2B. For the seven samples before anti-cancer treatment, the concordance rates were 33.3% and 75% in mutant *EGFR* and non-mutant *EGFR* groups, respectively. After three months of anti-cancer treatment, we did not detect any *EGFR* mutations in the CTC samples due to no CTC detected.

One incidental finding in this study was that there was one patient (1/80, 1.25%, patient #14) who had a de novo *T790M* mutation, which was compatible with the tissue findings. Another patient (1/80, 1.25%, patient #28) had an acquired *T790M* mutation after treatment with EGFR TKI, which was detected by the CTC *EGFR* test. Supplementary Figure S3A displays representative electrophoresis agarose gel after WGA. All the samples were sent for Sanger sequencing. One patient harboring both *L858R* and *T790M* (both cancer and PS-CTCs) is shown in Supplementary Figure S3B. The theoretical low limit of detection was one cell with approximately only 5–10 pg DNA.

### 2.3. The Lowest Detection Limit for EGFR Mutation and Quality Controls

We tested the lowest detection limits of the CTC *EGFR* mutational testing platform. The lowest detection limits are listed in Supplementary Table S2. The mutant DNA linear detection range in a total of 10 ng background non-mutant DNA ranged from 50 pg to 1 ng mutant DNA. The results showed that the detection limit of exons 18 and 20 was 50 pg in ARMS PCR, which was a better performance than 500 pg in the TaqMan^®^ platform.

Although challenging, the detection limit of 19 deletions in liquid biopsy using a non- next-generation sequencing (NGS) platform (only tested in TaqMan^®^ platform) in this study was 200 pg ctDNA (Supplementary Table S3). Meanwhile, DNA quality and linear amplification for rare cell PCR were qualified simultaneously.

To ensure the purity and efficiency of CTC isolation using the platform proposed in this study, we collected 4 mL of whole blood samples from healthy enrolled individuals (n = 5) in cell-line spiking experiments. The efficiency showed a mean ± SD of 97.4 ± 4.6% (ranging from 89.3% to 100.0%, with target cells ranging from 7 to 115 cells).

## 3. Discussion

We investigated whether high-purity CTCs can be captured from patients with NSCLC for *EGFR* mutational testing by combining negative and positive selection methods followed by On-Chip Sort (Figure 2 and Figure 3). The obtained CTCs showed high concordance (88.2%, Table 2) with tissue *EGFR* mutation detection in a highly efficient manner (Supplementary Table S4). In this exploratory study (28 NSCLC patients with 80 CTC samples, Table 1 and Table 2, and Figure 1), these novel methods yielded high specificity (90.9%), sensitivity (88.2%), and positive (93.8%) and negative (83.3%) predictive values (Table 2). Our results are comparable to findings in the literature. A study used CellSearch to capture CTCs and detected mutation type by NGS and revealed 94% concordance with tumor tissue [28]. In a meta-analysis that included 25 studies, circulating free DNA (cfDNA) for *EGFR* detection showed overall specificity (0.90), sensitivity (0.61), and concordance rates (0.79) [29]. In clinical practice, NGS and cfDNA detection are expensive and time-consuming. In our study design, CTCs on-chip sorting and ARMS and TagMan PCR to detect *EGFR* mutation could decrease the cost and save time, thereby increasing the clinical application.

In this prospective study, we defined CTCs as cells enriched from patients’ peripheral blood with expressions status of EpCAM^+^TTF-1^+^CD45^−^Hoechst^+^. Among all the markers, EpCAM has been referred to as a universal molecular marker for the detection of circulating tumor cells (CTCs) by the CellSearch system (Veridex, Warren, NJ, USA) [30]. The positive definition of a single CTC varies in the literature [31]. The most commonly used surface markers included EpCAM, E-cadherin, cytokeratins (Cks), Zonula occludens (ZO), and epithelial splicing regulator1 (ESPR1), or Vimentin [31,32]. Our study applied a similar panel to previously published studies [33,34,35]. Specifically, in some studies, cancer cells harboring *EGFR* mutations seemed to have a higher level of EpCAM expression, supporting the strategy of using the current panel to isolate CTC for *EGFR* mutational testing in our study [36].

Our study provided 87% sensitivity, 100% specificity, 100% positive predictive values (PPV), and 71% negative predictive value (NPV) for CTC *EGFR* mutation detection (Table 2). The main reason for this may be that we used a combination of negative and positive selection strategies. The reasons included: (i) a negative-only selection protocol can cause a low purity of *EGFR*-mutant cells in the sample, resulting in a false-negative finding; (ii) a positive-only selection protocol can cause a low recovery rate of CTCs at the beginning of CTC enrichment, resulting in false-negative findings in the following steps; (iii) CTCs with epithelial-mesenchymal transition (EMT) characteristics might have no or weak expression of EpCAM, causing difficulty in capturing CTCs to perform *EGFR* testing [37,38]. Given the complex biology of CTCs in a patient with advanced cancer, combining a negative and a positive selection strategy can maintain the recovery rate and purity of isolated CTCs before *EGFR* testing.

Our data showed that detection of *EGFR* mutations failed in CTCs of many patients when they were receiving anti-cancer therapy (at the third-month timepoint, Table 2). This phenomenon is consistent with earlier findings [39,40]. As early as 2008, Maheswaran S, et al. noticed that 4 of 27 patients with NSCLC after 3 months of EGFR TKI therapy had significantly lower numbers of CTCs captured by a microfluidic device than that in the samples before therapy [39]. Moreover, Iwama E et al. reported that the detection of *EGFR* mutation after 4 and 24 weeks of EGFR TKI therapy was 13.3% and 0%, respectively, from 19 patients with mutant NSCLC [40]. These results support our finding that the *EGFR* detection sensitivity in patients after three months of EGFR TKI therapy is relatively low. In our study, the low detection rate of *EGFR* mutation in CTCs might be secondary to the reduced number of CTCs isolated. After anti-cancer therapy, patients had low CTC detection, indicating a successful suppression of CTC release from primary lung cancer to foresee an upcoming disease control by standard imaging evaluation [41,42,43,44].

The present study has several limitations. First, this prospective study enrolled only a limited number of patients. Although the case number is relatively small, a sensitivity of 87%, a specificity of 100%, PPV of 100%, and NPV of 71% for CTC *EGFR* mutation detection (Table 2) are acceptable compared with that of ctDNA (a pooled sensitivity for cfDNA to detect *EGFR* mutations of 61% and 67.4% with a specificity of 90% and 93.5%) [29,45,46]. Second, the optimal timing of CTC testing after therapy remains unclear in this study. This might be related to a reasonable disease control rate and a long PFS when treated with EGFR TKI. More tests to monitor the *EGFR* mutational status using CTCs until disease progression may be a good design in the future.

In conclusion, we report that CTCs captured by negative and positive selection followed by the on-chip sort platform represent a suitable and reliable source of lung cancer tumor DNA for detecting *EGFR* mutations, including T790M. This novel diagnostic method could be helpful in obtaining an *EGFR* mutation status in cases with inadequate tissue or intolerance to the invasive re-biopsy procedure. This approach could also be beneficial for monitoring treatment response and real-time tumor genotyping.

## 4. Materials and Methods

### 4.1. Patient Enrollment

We invited patients who visited the Chang Gung Memorial Hospital between July 2016 and September 2018 to participate in the study. Patients were considered eligible if they fulfilled the followings criteria: (1) 20 years of age or older, (2) newly diagnosed and treatment naïve stage IV lung adenocarcinoma based on the 8th edition of the American Joint Committee on Cancer, (3) unknown *EGFR* mutation status but under evaluation, and (4) recurrent lung cancer that received standard treatment. The exclusion criteria were (1) synchronous cancer or previous cancers within the last five years; (2) refusal of blood drawing, (3) no final tissue *EGFR* mutation result, and (4) less than three months of survival time. The study received institutional review board approval (approval IDs: 104-9796B, 201509796B0C601, and 201702296B0). Informed consent was obtained from each patient before enrollment, in accordance with the Declaration of Helsinki. The National Ministry of Health and Welfare’s guidelines for the care and use of cancer cell lines and human samples were strictly followed. After blood sampling, the patient received anti-cancer therapy according to the *EGFR* mutation results and Taiwan’s standard lung cancer treatment guidelines.

### 4.2. Specimen Collection, Delivery, and Processing

When pathology confirmed lung adenocarcinoma, we checked for *EGFR* mutation from tissue specimens and concurrently performed *EGFR* mutation tests in CTCs from the blood at diagnosis. Pathologists analyzed *EGFR* mutation from tissue specimens using amplification-refractory mutation system (ARMS) analysis or TaqMan™ system at Linkou Chang Gung Memorial Hospital. After three months of systemic anti-cancer treatment, a second CTCs test was performed for longitudinal follow-up purposes, as designed.

### 4.3. CTC Isolation and Quality Controls

The blood of the patients was collected and sent to the laboratory within 48 h for CTC extraction. Target cells were extracted from a fresh blood sample using a negative selection method. We then sent the negatively selected CTCs (NS-CTCs) to the On-Chip Sort machine (On-Chip Biotechnologies, Tokyo, Japan) and isolated the positively-selected CTCs (PS-CTCs)**.** We used the On-Chip Flow Ver1.8.12 software for all the cell analyses. PS-CTCs are defined as cells expressing both EpCAM and Hoechst. The details of the CTC isolation protocols are detailed in the Supplementary Materials and methods, including thyroid transcription factor-1 (TTF-1) and EpCAM expression.

To determine the purity of the PS-CTCs, H1975 cells (#CRL-5908, ATCC, VA, USA) were pre-stained with CellTrace™ Calcein Red-Orange (C34851, Thermo Scientific, MA, USA) at room temperature (RT) for 30 min. After washing twice, the cells were serially diluted and counted for the subsequent spiked tests. The recovered positively selected cells were estimated under a phase-contrast fluorescence inverted microscope (Axiovert 200 M, Carl Zeiss, Jena Deutschland). The purity was defined as the target/(target cell + non-target cell) ratio. Representative IF images of TTF-1-expressing CTCs were obtained using a phase-contrast fluorescence inverted microscope (Axiovert 200 M, Carl Zeiss, Jena Deutschland, Supplementary Figure S1).

### 4.4. DNA Extraction from CTCs and Analysis

Two protocols were used to evaluate the *EGFR* mutations in CTCs. Genomic DNA was extracted from PS-CTCs using the QIAamp DNA Micro Kit (#56304, Qiagen, Hilden, Germany) and then processed by whole-genome amplification (WGA) using the REPLI-g Single Cell kit (#150345, Qiagen, Hilden, Germany). For exploratory purposes, ARMS PCR was added to the procedure. We analyzed *EGFR* mutations (exon 18 *G719S/G719C*, exon 20 *T790M,* and exon 21 *L858R)* by ARMS PCR using the GoTaq qPCR Master Mix (#A6001, Promega, WI, USA). For the positive control of *EGFR* mutations, SW48 cells (#CCL-231, mutant *EGFR* at exon 18 G719S, ATCC, VA, USA) and H1975 cells (#CRL-5908, harboring both exon 20 *T790M* and exon 21 *L858R* mutations in *EGFR*, ATCC, VA, USA) were used. WBCs from healthy donors were used as the negative control. Details of the PCR sequences are presented in Supplementary Table S1. The brief mechanism of detection is displayed in Supplementary Figure S2. All PCR data were analyzed using an ABI 3730xl DNA Analyzer (Applied Biosystems, CA, USA) and ChromasPro 2.1.8 software (Technelysium Pty Ltd., QLD, Australia). Mutated *EGFR* was confirmed by the Sanger sequencing.

In addition to ARMS PCR, we attempted to develop parallel testing using the TaqMan^®^ Mutation Detection Assays to improve the turnaround time of CTC *EGFR* testing.

### 4.5. Statistical Analysis

The patients’ demographic data were summarized as the number (%) for categorical variables and the median, 95% confidence interval (CI), and range for continuous variables. Descriptive statistics were used for the basic characteristics. SPSS software (version 24.0; SPSS Inc., Chicago, IL, USA) was used for all the statistical analyses.

## Figures and Tables

**Table 1:** Basic characteristics of enrolled patients and samples (n = 28).

Characteristics	n	%
Age, years	Mean (median, range) = 57.4 (56, 36–79)	
Ethnicity	Asian	100.0%
Sex		
Male	12	42.9%
Female	16	57.1%
Adenocarcinoma	28	100.0%
T category		
1–2	11	39.3%
3–4	17	60.7%
N category		
0–1	10	35.7%
2–3	18	64.3%
M category		
1a	2	7.1%
1b	12	42.9%
1c	14	50.0%
Overall Staging (AJCC 8th edition)		
Stage IVa	12	42.9%
Stage IVb	16	57.1%
Sites of distant metastasis		
Contralateral lung (M1a)	14	50.0%
Bone	13	46.4%
Brain	10	35.7%
Distant lymph node	7	25.0%
Liver	7	25.0%
Pleura	4	14.3%
Adrenal gland	3	10.7%
Tissue *EGFR* mutations		
E19 Deletion	11	39.3%
L858R mutation	5	17.9%
Synchronous T790M and L858R mutations	1	3.6%
Non-mutant	11	39.3%

Abbreviations: *EGFR*, Epidermal growth factor receptor; AJCC, American Joint Committee on Cancer.

**Table 2:** The concordance of *EGFR* mutations between circulating tumor cells and cancer tissues.

	N	CTC-DNA for *EGFR*		Concordance of *EGFR* Mutations of CTCs
		*EGFR* ^mutant^	*EGFR* ^non-mutant^	
**A. EGFR testing (n = 80)**				
Before treatment *	28	16	12	
Tissue *EGFR*^mutant^	17	15	2	88.2%
Tissue *EGFR*^non-mutant type^	11	1	10	90.9%
**Under treatment at 3 M after treatment**	**52**	**1**	**51**	
Tissue *EGFR*^mutant^ #	30	0	30	0.0%
Tissue *EGFR*^non-mutant type^	22	1	21	95.5%
**B. EGFR testing using ARMS-PCR (n = 12)**				
**Before treatment (n = 7)**	**7**	**4**	**3**	
Tissue *EGFR*^mutant^	3	1	2	33.3%
Tissue *EGFR*^non-mutant^	4	3	1	75.0%
**Under treatment (n = 5) at 3 M after treatment**	**5**	**0**	**5**	
Tissue *EGFR*^mutant^	5	0	5	0.0%
Tissue *EGFR*^non-mutant^	0	0	0	NA

* Among patients before treatment, the accuracy, sensitivity, specificity, positive predictive value, and negative predictive values of the *EGFR* mutation tests on CTCs are 89.3%, 88.2%, 90.9%, 93.8%, and 83.3%, respectively. # These patients all received standard EGFR tyrosine kinase inhibitors, including afatinib, gefitinib, or erlotinib.

## Data Availability

Not applicable.

## Supplementary Materials

The following supporting information can be downloaded at: https://www.mdpi.com/article/10.3390/ijms231810661/s1.
