# Supplementary material for: Circulating EGFR Mutations in Patients with Lung Adenocarcinoma by Circulating Tumor Cell Isolation Systems: A Concordance Study
Source: Int J Mol Sci. 2022 Sep 13;23(18):10661. doi: 10.3390/ijms231810661 (PMC9505961; doi:10.3390/ijms231810661)
Supplement: Supplementary file 1 [file ijms-23-10661-s001.zip › ijms-1863838-supplementary/Supplementary Tables 04Jun2022.pdf]

Supplementary Tables

Negative selection of circulating tumor cells

The whole CTC isolation protocol was based on a successful negatively selected (NS)-CTC. In brief, the platform can have a 44.6±9.1% and a coefficient of variation of 20.4%. For the brief protocol description, we drew 4 milliliters (mL) of peripheral blood into EDTA vacuum tubes first. To remove red blood cells, 4 mL of whole blood was incubated with RBC lysis buffer (#20120, StemCell Technologies, BC, Canada) for 5 mins at room temperature (RT) and centrifuged at 400g for 10 mins. We removed the supernatant and washed the cell pellet with wash buffer (RPMI-1640 medium supplemented with 10% fetal bovine serum, Thermo Scientific, MA, USA) twice. The cell pellet was then incubated with a CD45-depletion kit (#18259, StemCell Technologies, BC, Canada) and an Anti-Human Glycophorin depletion kit (#18352, StemCell Technologies, BC, Canada) at RT for 10 mins. We followed the kit illustrations to suspend the cell mixture with 2 mL wash buffer and incubate for 15 mins in Magnet set (#18000, StemCell Technologies, BC, Canada). After the supernatant was discarded, we transferred the remnants to a new tube and centrifuged the sample with 400g for 10 mins. NS-CTCs were enriched after the removal of supernatant

For further identification and confirmation, the NS-CTCs were further stained with Alexa Fluor 488-conjugated mouse anti-human epithelial cell adhesion molecule (#5198S, EpCAM, 1: 200 dilutions, Cell Signaling, MA, USA) and Hoechst 33342 solution (#62249, 1: 200 dilution, Thermo Scientific, MA, USA) at RT for 1 hr. After being washed three times, the cell mixture was fixed in fixation medium A (#GAS001S5, Thermo Scientific, MA, USA) for 15 min at RT. The samples were further washed three times in ESB buffer (#10010031, PBS-Phosphate Buffered Saline, Thermo Scientific, MA, USA) supplemented with FBS (#31606-01, Fetal Bovine Serum, qualified, One Shot™ format, USDA-approved regions, Thermo Scientific, MA, USA) and Ethylenediaminetetraacetic acid (#E9884, EDTA, Sigma-Aldrich, MO, USA) at a final concentration of 10% and 100 mM, respectively. Then, the samples were incubated in permeabilization medium B (#GAS002S100, Thermo Scientific, MA, USA). The fixed and permeabilized cells were further separately stained with rabbit anti-human thyroid transcription factor-1 (TTF-1) primary antibody (1: 100 dilution, Abcam Biochemicals, Cambridge, UK) and normal rabbit IgG isotype control (#NB810-56910, 1: 100 dilution, Novus Biologicals, Colorado, USA) for 20 min at RT. The samples were subsequently washed in PBS three times. Alexa Fluor 647-conjugated goat anti-rabbit secondary antibody (#ab150079, 1: 1000 dilution, Abcam Biochemicals, Cambridge, UK) was added to the samples. TTF-1 identification on CTCs was confirmed by flow cytometry (CytoFLEX flow cytometry with CytExpert cytometry analysis software version 2, Beckman Coulter, GA, USA).

Table S1. Sequences of primer sets for two PCR method

A. For Sanger sequences	
G719S/C -forward	5'-TTCCAAATGAGCTGGCAAGT-3'
G719S/C -reverse	5'-TGCCTTTGGTCTGTGAATTG-3'
T790M -forward	5'-CGCATTCATGCGTCTTCACC-3'
T790M -reverse	5'-CTATCCCAGGAGCGCAGACC-3'
L858R -forward	5'-GTCAGCAGCGGGTTACATC-3'
L858R -reverse	5'-ATCCTCCCCTGCATGTGTTA-3'
E19Del -forward	5'-CGTCGCTATCAAGACATCTC-3'
E19Del -reverse	5'-GGCCAGTGCTGTCTCTAAGG-3'
B. For ARMS-PCR	
G719S/C-forward	5’-CAAAAAGATCAAAGTGCTGW-3’
G719S/C-reverse	5’-TAACTTGGGAAAAACACTGG-3’
T790M -forward	5’-TCCACCGTGCAGCTCATCAT-3’
T790M -reverse	5’-GCTATCCCAGGAGCGCAGAC-3’
L858R -forward	5’-AGATCACAGATTTTGGGCG-3’
L858R-reverse	5’-GCTCACACTACCAGGAGA-3’

Table S2. The lower detection limits for *EGFR* mutations by real-time PCR, compared with a commercial TaqMan® qPCR system.

	Exon 18		Exon 19	Exon 20		Exon 21	
	**ARMS PCR**	**TaqMan®**	**TaqMan®**	**ARMS PCR**	**TaqMan®**	**ARMS PCR**	**TaqMan®**
NC	-	-	-	-	-	-	-
10ng W	42.9 ± 0.7	-	-	36.8 ± 0.3	-	-	-
50pg M	40.7 ± 1.4	-	31.8 ± 0.4	33.7 ± 0.2	-	38.3 ± 1.0	30.4 ± 0.2
100pg M	39.0 ± 0.8	-	30.7 ± 0.6	32.9 ± 0.3	-	37.5 ± 0.2	29.6 ± 0.2
500pg M	36.4 ± 0.7	32.6 ± 0.3	28.3 ± 0.2	29.9 ± 0.0	31.4 ± 1.6	34.8 ± 0.2	26.5 ± 0.2
1ng M	34.8 ± 0.7	31.5 ± 0.2	27.3 ± 0.2	28.7 ± 0.4	32.2 ± 0.5	34.0 ± 0.3	25.9 ± 0.1
0.5% M (50pg M + 10ng W)	40.8 ± 2.6	-	31.1 ± 0.5	33.9 ± 0.1	-	38.5 ± 0.4	29.7 ± 0.2
1% M (100pg M+ 10ng W)	38.3 ± 1.6	-	30.7 ± 0.3	32.6 ± 0.2	-	37.7 ± 0.2	29.1 ± 0.4
5% M (500pg M +10ng W)	35.8 ± 1.1	32.4 ± 0.7	28.3 ± 0.3	29.8 ± 0.2	32.9 ± 0.5	34.8 ± 0.1	26.4 ± 0.1
10% M (1ng M +10ng W)	34.4 ± 0.8	31.8 ± 0.2	27.2 ± 0.1	28.7 ± 0.4	32.7 ± 1.0	33.6 ± 0.5	25.4 ± 0.2

Abbreviations: NC, negative control; W, gDNA of white blood cells from health donors as a control for *EGFR*$^{wild\ type}$ M, gDNA from cell line of SW48 (G719S mutation in exon 18), H1975 (T790M in exon 20, L858R in exon 21) and H1650 (19 deletion) as the controls for *EGFR*^{mutant}, respectively. Data was presented using the mean of Ct mean ± SD of three individual experiments; Ct, threshold cycle of qPCR. The "-" indicates undetectable *EGFR* mutation status.

Table S3. Qualified the DNA quality and linear amplification for rare cell PCR

	E19_Reference Assay	
	Ct	SD
NC	-	-
200pg WT	30.0	0.3
2ng WT	26.2	0.2
20ng WT	22.8	0.1

Abbreviations: E19_Reference Assay, TaqMan® Mutation Detection Reference Assays for EGFR Exon 19. Ct, means of threshold cycle of qPCR. SD, standard deviation. Data represent the mean of Ct (± SD) of three individual experiments.

Table S4. Comparisons between two protocols to detect *EGFR* mutations on circulating tumor cells

	PS-CTC WGA	PS-CTC ARMS PCR
**CTCs purity before PCR**	90~100%	90~100%
**Detection methodology**	Sanger sequencing	ARMS-PCR
**Unknown mutant site detectability**	Yes	No
**Mutant specific amplification**	No	Yes
**Lowest mutant DNA proportion**	15~20%	~0.5%
**Time costs per case**	3~4 days	~8 hours
**Limitation**	Inevitable amplification error	Limited amount of DNA for multiple gene analysis

Time costs per case, all procedures from whole blood to data output.
Abbreviations: PS-CTC, modified positively selected circulating tumor cell; ARMS-PCR, Amplification refractory mutation system PCR; WGA, whole-genome amplification
