# Supplementary figures and images for: Circulating EGFR Mutations in Patients with Lung Adenocarcinoma by Circulating Tumor Cell Isolation Systems: A Concordance Study
Source: Int J Mol Sci. 2022 Sep 13;23(18):10661. doi: 10.3390/ijms231810661 (PMC9505961; doi:10.3390/ijms231810661)

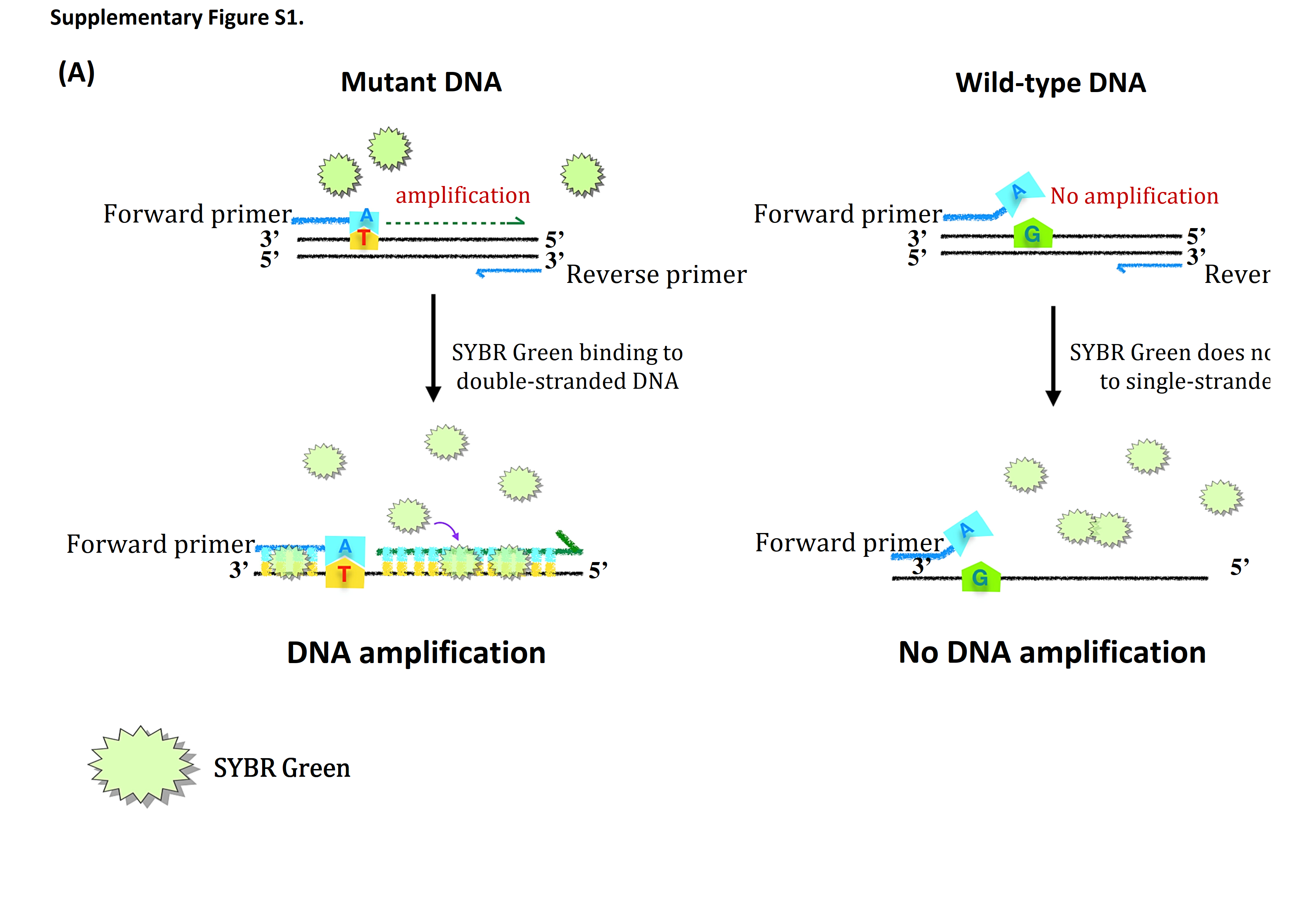

Supplement: Supplementary file 1 [file ijms-23-10661-s001.zip › ijms-1863838-supplementary/Sup Fig S2A ARMS ttf.tif]

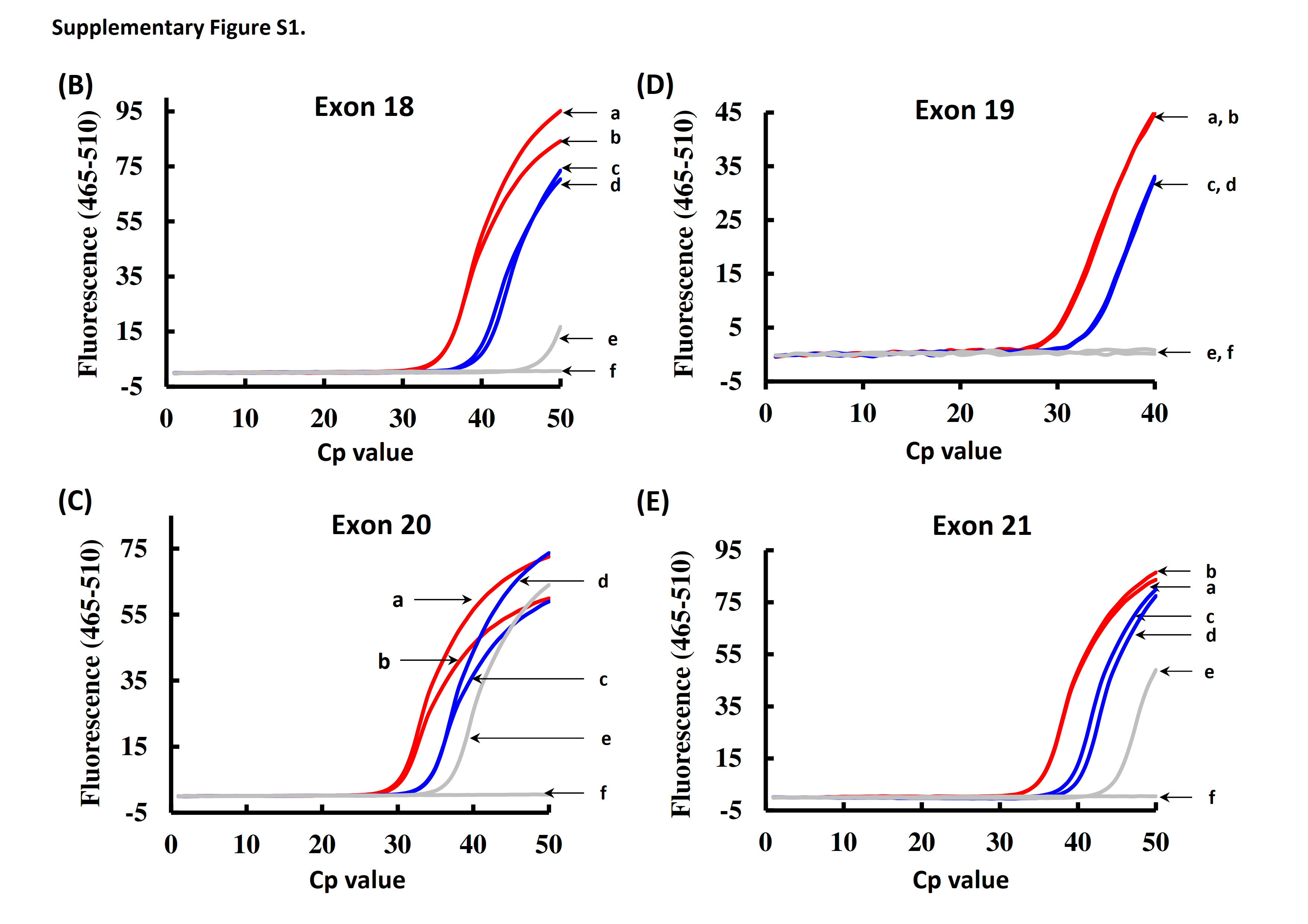

Supplement: Supplementary file 1 [file ijms-23-10661-s001.zip › ijms-1863838-supplementary/Sup Fig S2BCDF ARMS ttf.tif]

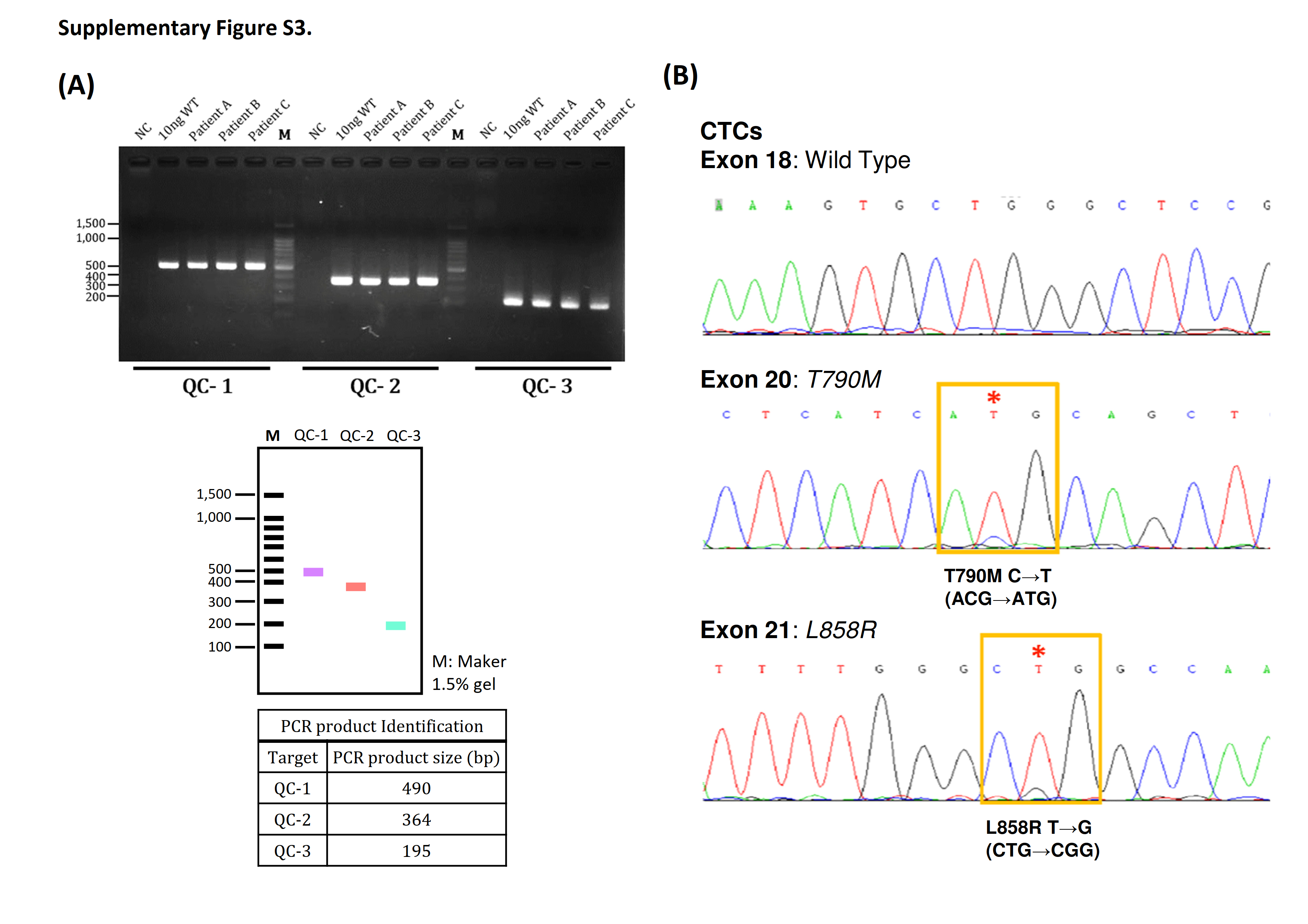

Supplement: Supplementary file 1 [file ijms-23-10661-s001.zip › ijms-1863838-supplementary/Sup Fig S3 PCR sequencing ttf.tif]

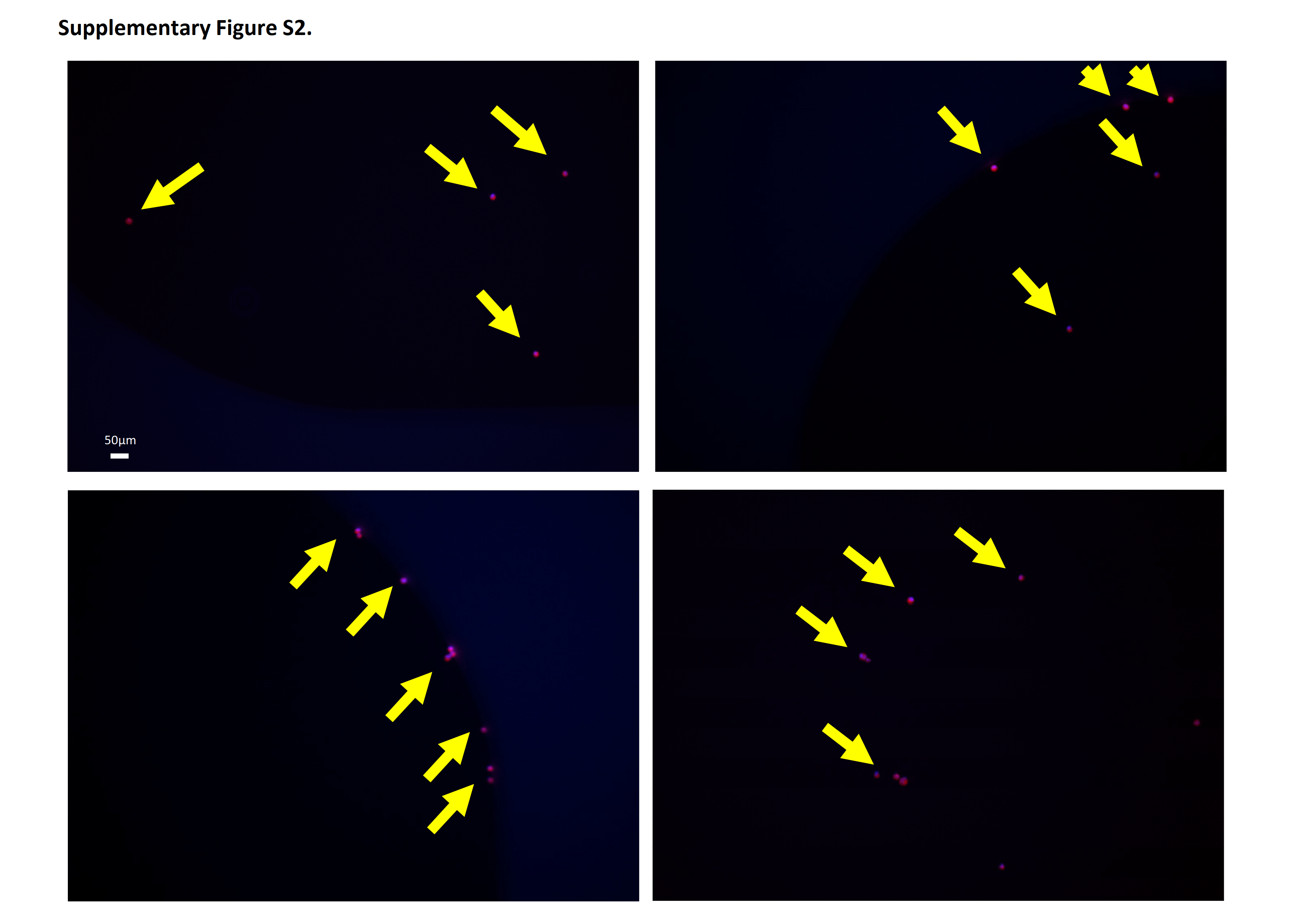

Supplement: Supplementary file 1 [file ijms-23-10661-s001.zip › ijms-1863838-supplementary/Sup Figure S2 IFC CTCs ttf.tif]
